# Cognitive Implications of Correlated Structural Network Changes in Schizophrenia

**DOI:** 10.3389/fnint.2021.755069

**Published:** 2022-01-20

**Authors:** Dawn M. Jensen, Elaheh Zendrehrouh, Vince Calhoun, Jessica A. Turner

**Affiliations:** ^1^Neuroscience Institute, Georgia State University, Atlanta, GA, United States; ^2^Department of Computer Science, Georgia State University, Atlanta, GA, United States; ^3^Tri-Institutional Center for Translational Research in Neuroimaging and Data Science (TReNDS), Georgia State University, Georgia Institute of Technology, Emory University, Atlanta, GA, United States; ^4^Department of Psychology, Georgia State University, Atlanta, GA, United States

**Keywords:** multimodal imaging, cognition, gray matter density, fractional anisotropy, schizophrenia

## Abstract

**Background:**

Schizophrenia is a brain disorder characterized by diffuse, diverse, and wide-spread changes in gray matter volume (GM) and white matter structure (fractional anisotropy, FA), as well as cognitive impairments that greatly impact an individual’s quality of life. While the relationship of each of these image modalities and their links to schizophrenia status and cognitive impairment has been investigated separately, a multimodal fusion *via* parallel independent component analysis (pICA) affords the opportunity to explore the relationships between the changes in GM and FA, and the implications these network changes have on cognitive performance.

**Methods:**

Images from 73 subjects with schizophrenia (SZ) and 82 healthy controls (HC) were drawn from an existing dataset. We investigated 12 components from each feature (FA and GM). Loading coefficients from the images were used to identify pairs of features that were significantly correlated and showed significant group differences between HC and SZ. MANCOVA analysis uncovered the relationships the identified spatial maps had with age, gender, and a global cognitive performance score.

**Results:**

Three component pairs showed significant group differences (HC > SZ) in both gray and white matter measurements. Two of the component pairs identified networks of gray matter that drove significant relationships with cognition (HC > SZ) after accounting for age and gender. The gray and white matter structural networks identified in these three component pairs pull broadly from many regions, including the right and left thalamus, lateral occipital cortex, multiple regions of the middle temporal gyrus, precuneus cortex, postcentral gyrus, cingulate gyrus/cingulum, lingual gyrus, and brain stem.

**Conclusion:**

The results of this multimodal analysis adds to our understanding of how the relationship between GM, FA, and cognition differs between HC and SZ by highlighting the correlated intermodal covariance of these structural networks and their differential relationships with cognitive performance. Previous unimodal research has found similar areas of GM and FA differences between these groups, and the cognitive deficits associated with SZ have been well documented. This study allowed us to evaluate the intercorrelated covariance of these structural networks and how these networks are involved the differences in cognitive performance between HC and SZ.

## Introduction

Schizophrenia is a complex and chronic brain disorder that has severe negative impact on an individual’s daily life. The disorder is often diagnosed after an initial psychotic episode in late adolescence to early adulthood (Picchioni and Murray, 2007). This is generally preceded by a prodromal period during which there are subacute changes in general thinking, mood, and social functioning (Picchioni and Murray, 2007). Symptoms of schizophrenia fall into one of three categories: positive, negative, and cognitive. Positive symptoms are generally considered to include hallucinations, delusions, and disordered thinking. Negative symptoms are seen as flattened affect, anhedonia, and apathy (Picchioni and Murray, 2007). Cognitive symptoms manifest in problems with attention, concentration, and memory (Green and Harvey, 2014). The combined impact of these impairments greatly diminish the quality of life experienced by patients with schizophrenia throughout their lifetime. There is no known cure, despite schizophrenia’s prevalence of roughly 1% of the population, although anti-psychotic and anti-depressant medications mitigate some of the positive and negative symptoms.

The cognitive deficits associated with schizophrenia are considered one of its core dysfunctions. The degree of impairment is diverse within the disease, impacting many cognitive functions such as sensory processing, inhibition, attention, language function, working memory, episodic memory, and executive function; and it may be also a better predictor of functional recovery and overall outcome than any of the other symptoms patients with schizophrenia suffer (Green and Harvey, 2014). The source of these cognitive deficits is thought to be the pervasive changes in gray and white matter that occur in the transition from prodromal through the first episode of psychosis. These stabilize post-first episode and generally do not decline over the remainder of the illness. The diverse changes to these brain structures disrupt the cortical-subcortical-cerebellar circuit within the brains of patients with schizophrenia, negatively impacting a large number of cognitive domains (Barch and Ceaser, 2012). Because the cognitive deficits are so wide and diverse across the various cognitive domains, we chose to consolidate the cognitive test used in this study into a universal measurement of cognitive performance (g) (Gottfredson, 1998). Neuroscience and neuroimaging studies have shown over time that most higher cortical functions (such as cognition) are distributed widely over the entire brain, rather than localized to particular regions (Barrett and Satpute, 2013). While some regions may be more involved in a particular process than another, there are very few processes that only involve discreet structures of the brain. This understanding of the networked nature of cognition has driven the interest in whole brain analysis to further our understanding of the interconnected interplay (networks) of the entire brain, rather than focusing on specific and isolated structures.

Neuroimaging studies of the disease have shown it to be characterized by wide spread changes in the gray matter volume and the integrity of white matter structures. A large-scale international ENIGMA Schizophrenia Working Group study found that the entire brain showed reduced cortical thickness in patients. Some of the largest effects were seen in the superior temporal gyrus, superior frontal gyrus, middle frontal gyrus, precuneus, and cerebellum (Gupta et al., 2015). A 2016 ENIGMA study also found several subcortical regions in the brain, including the hippocampus, thalamus, and amygdala, had smaller gray matter volumes in patients (van Erp et al., 2016). Smaller studies have commonly found that patients with schizophrenia to also have reduced gray matter volume in subcortical areas such as the hippocampus and thalamus (Bora et al., 2011; Shepherd et al., 2012). Several studies have shown that these areas of reduced gray matter volume have been related to the diminished cognitive performance associated with the disorder. In a study of first-episode patients, the researchers found that cognitive deficits were strongly correlated with the reduced prefrontal and temporo-parietal GM (Minatogawa-Chang et al., 2009). The changes to white matter structure found in schizophrenia are just as profound and pervasive. An ENIGMA study of white matter integrity showed global reduction in FA, with the largest effects seen in the anterior corona radiata, the entirety of the corpus callosum, the cingulum, and the posterior thalamic radiation (Kelly et al., 2018). This is also in keeping with a 2017 review of DTI studies in schizophrenia that shows reduced white matter structural integrity in nearly every tract of the brain (Parnanzone et al., 2017). Smaller studies have shown relationships between lower white matter integrity in patients with schizophrenia and impaired cognitive performance in cognitive domains such as working memory (Karlsgodt et al., 2008; Epstein et al., 2014).

A recent study in animals has shown that MRI GM volume reflects not only the physical volume of the dendrites (gray matter), but also the glial and other support cells, as well as the clustering behaviors of the dendrites (Asan et al., 2021). These changes in the cellular composition within the gray matter have been linked to cognitive performance, although the exact mechanism underlying this is not fully understood. Other studies in animals show that changes in dendritic health and the composition of the glial cells associated with them impair neuroplasticity, which then impairs cognitive performance (Vance et al., 2010; Forrest et al., 2018). Disruptions in white matter integrity are thought to impact cognitive performance on the cellular level due to less stable flow of electrical currents, disruptions of the conduction of action potentials, which then lead to fluctuations in the connectivity of neuronal pathways, reduced efficacy of neurotransmitter systems, and disconnectivity in associative pathways (Fjell et al., 2011). Schizophrenia has been understood to develop from changes within the gray and white matter, as a combination of genetic and environmental factors, that lead to positive and negative symptoms as well as the cognitive impairment associated with it (Bora, 2015).

To date, these structural networks and their relationships to cognition have only been studied unimodally. Newer multimodal analysis tools allow us to consider the simultaneous pattern of these two networks in way previously not possible. The unimodal studies have shown which structural (GM or FA) brain regions differ, but they are limited in that they cannot evaluate the direct relationship between GM and FA. Multimodal studies quantify the simultaneous relationships of different brain measures in ways that are not possible separately. This study uses a multimodal analysis of magnetic resonance imaging (MRI), to examine how the pattern of simultaneous covariance of gray and white matter changes differ between healthy controls (HC) and patients with schizophrenia (SZ) and the relationship of those changes have with cognitive performance.

Parallel independent component analysis (pICA), a semi-blind multimodal analysis tool, of structural MRI (sMRI) and diffusion MRI (dMRI) is a method that highlights the correlated covariance of these structural networks (gray matter and white matter) in the brain (Sui et al., 2012). This data-driven method allows a whole brain exploration of the relationship between gray matter volume, measured with the sMRI, and the integrity of the white matter, reflected in the fractional anisotropy (FA), measured using dMRI (Calhoun and Sui, 2016). pICA uses independent component analysis (ICA) to identify the maximally independent components of both modalities while simultaneously estimating the degree of correlation between them (Pearlson et al., 2015). pICA can be used to both identify and quantify the relationships between the features, or spatial maps, within the structural networks of gray and white matter. Variance between individual subjects is reflected in the loading coefficients of each feature (Calhoun and Adali, 2009).

A previous multimodal study using joint ICA (jICA) did identify group differences within joint sources of gray and white matter volume between healthy controls (HC) and patients with schizophrenia (SZ) (Xu et al., 2009). This study considered white matter volume, rather than looking at the integrity of white matter structure as reflected in FA. More recently, multimodal studies also used a jICA of GM, FA, and the fractional amplitude of low-frequency fluctuation (fALFF) of patients with SZ, combined with a reference map derived from a multimodal canonical correlation analysis (MCCAR) of cognitive performance, to develop replicable neural markers of the disease (Sui et al., 2013, 2015, 2018). Those studies used spatial maps from a template derived from cognitive performance to predict changes in a jICA of those three modalities, which is the reverse of our approach. Here, we identify correlated GM and FA patterns that are affected by diagnostic status, and then consider the relationship of those components with cognitive function. jICA, while also a fusion analysis technique, differs from pICA considerably. A jICA assumes that the signal sources (for example GM, FA, falff, and scores from specific cognitive domains) will all modulate the same way across the subjects (Calhoun and Adali, 2009). This strong constraint simplifies the estimation of joint information, but does not allow for differentiated covariation between signal sources. pICA does not make this assumption and allows each signal source (in this case, GM and FA) to vary independently and then optimizes the correlated patterns of covariation found between them (Calhoun and Adali, 2009). pICA thus uses a “soft” constraint, whereas jICA uses a “hard” constraint on the inter-modality coupling.

## Materials and Methods

### Subjects

The images were collected from 157 subjects, 82 healthy controls (HC) and 75 subjects with schizophrenia (SZ) as part of the COBRE dataset, collected according to the description in Aine et al. (2017). Two subjects with schizophrenia were removed, one for failing the diffusion tensor imaging (DTI) quality control (insufficient number of good gradients), the other for exceeding allowed motion parameters (more than 3 mm). The final sample total was 155, with the HC group comprising 62 males and 20 females, and the SZ group 59 males and 15 females. Both groups ranged from 18 to 65 years in age. The Structured Clinical Interview for DSM Disorders (SCID) was used to gather diagnostic information and subjects were excluded if they had history of substance abuse or dependence within the last 12 months, severe head trauma with more than 5 min loss of consciousness, neurological disorders, or severe cognitive impairment. The range of IQ, as reflected in the WASI Sum IQ metric, was from 65 to 134. Medication dose was calculated according to the methods outlined by Gardner et al. in their 2010 paper, *International Consensus Study of Antipsychotic Dosing* (Gardner et al., 2010). All subjects provided informed consent prior to the study. A Welch’s two sample *t*-test and a Pearson’s chi-squared test were used to test for group differences in age and gender, respectively, using R version 3.5.0. A Mood’s median test was performed to determine group differences in education levels and occupation levels (see Table 1).

**TABLE 1:** Demographic Statistics.

	Schizophrenia	Healthy controls	*p*
N	73	82	—
Sex (%male)	75.34%	75.61%	0.54
Age (mean/range)	37.32/18–65	38.93/18–65	0.44
Education level (median/range)	4/2–8	4/2–8	0.24
Occupation level (median/range)	5/0–7	4/1–7	0.21

*A Chi-squared test showed no significant differences regarding sex in patients or healthy controls. A Welch’s two sample t-test also found no significant differences between the groups for age. A Mood’s median test showed no significant differences between the education and occupation levels between the groups.*

### Image Collection

The imaging data were collected on a Siemens 3T Trio TIM scanner at the Mind Research Network, Albuquerque, NM. The T1-weighted images for GM were collected in the sagittal plane, interleaved, multi-slice mode in a single shot with these parameters: TR/TE/TI = 2,530/[1.64, 3.5, 5.36, 7.22, 9.08]/900 ms, flip angle = 7*, FOV = 256 × 256 mm, slab thickness 176 mm, matrix 256 × 256 × 176, voxel size = 1 × 1 × 1 mm, number of echos = 5, pixel bandwidth = 650 Hz, total scan time = 6 min.

The DTI images for FA were collected using 30 gradient directions and five b = 0, for a total of 72 slices with a slice thickness of 2 mm (isotroptic resolution of 2 × 2 × 2 mm). FOV = 256 × 256 mm, TR/TE = 9,000 ms/84 ms, encoded A-P. Sequence bandwidth was 1,562 Hz/Px and echo spacing was 0.72 ms with an EPI factor of 128. For more details, see Aine et al. (2017).

### Image Processing

#### Diffusion MRI to Fractional Anisotropy

An FSL v5.0.10 pipeline was used to preprocess the DTI data (Smith et al., 2004). A quality control of the DTI images was done using DTIPrep to ensure that a minimum of 25 gradient directions for each subject were free of artifacts (Liu et al., 2010). Eddy current correction for gradient distortions and head motion were applied to the diffusion-weighted images (Andersson and Sotiropoulos, 2016), after which a brain extraction tool (BET) was used to remove non-brain tissue from the image (Smith, 2002). A diffusion tensor model was fitted to each voxel with DTIFIT (Smith, 2002), creating the fractional anisotropy images. All subjects’ FA data were then aligned into a common space using the non-linear registration tool FNIRT (Andersson et al., 2007a,b), which uses a b-spline representation of the registration warp field (Rueckert et al., 1999). Leaving the FA unsmoothed and in 1 × 1 × 1 MNI152 resolution eliminated spurious results due to partial voluming.

#### Structural MRI to Gr**a**y Matter Volume

The T1-weighted sMRI images were reoriented and registered to the MNI152 template and resampled to 2 mm × 2 mm × 2 mm. Using DARTEL in SPM12 (Ashburner and Friston, 2005), the non-brain tissues were stripped and the gray matter, white matter, and cerebral spinal fluid were segmented, leaving normalized, modulated, Jacobian-scaled gray matter images. A QA was performed to ensure that the images produced correlated with the template (*r* > 0.85), and were then smoothed by an 8 mm × 8 mm × 8 mm Gaussian kernel.

#### Parallel Independent Component Analysis

Parallel ICA was performed using the Fusion ICA Toolbox (FITv2.0a) using Matlab R2017b. The number of principle components for each modality were estimated using a minimum description length (MDL) in the FIT software (4 FA components and 52 GM components when estimated separately, 12 when combined) (Li et al., 2007). The descending trend of entropy was allowed to be –0.001 maximally. ICASSO software was used to ensure cluster stability by retesting each FastICA 10 times. The suggested default of applying the constraint algorithms, which control for over-and under-fitting the data, to the first six component pairs was used (Sui et al., 2018). Spatial maps were calculated and loading coefficients extracted that reflect the decomposition of the subject’s data. Z-scores of the spatial maps were thresholded at |z| > 3 to identify component clusters. Case vs. control differences in the loading coefficients for the correlated pairs of each of the GM/FA spatial maps were calculated using a two-sample *t*-test, Bonferroni-corrected for multiple tests. The Harvard-Oxford Cortical and Subcortical Structure Atlases were used to identify the gray matter regions. The JHU ICBM-DTI-81 White Matter Labels were used to identify the white matter regions, except where the pICA identified white matter regions outside the 81 tracts provided, in which case, the corresponding gray matter region was labeled as above.

#### Cognitive Performance

All individuals in the study were administered a battery of neuropsychological tests. All cognitive tests were collected within 1 week of every neuroimaging assessment (Aine et al., 2017). These included: the Continuous Performance Test-Identical Pairs (CPT-IP), the Neuropsychological Assessment Battery (NAB), the Measurement and Treatment Research to Improve Cognition in Schizophrenia (MATRICS), Wechsler Abbreviated Scale of Intelligence (WASI_IV), the Processing Speed Index of the Wechsler Adult Intelligence Scale_IV including symbol search and coding (WAIS_IV), and the Wechsler Test of Adult Reading (WTAR) (Aine et al., 2017). Data reduction was performed using a principal component analysis (PCA) using R version 3.5.0, of which the first component reflects a composite of the cognitive performance (g).

### Statistical Analyses

A multiple analysis of covariance (MANCOVA) was performed using R version 3.5.0 to test the significance of the relationships between the loading coefficients identified by the pICA spatial maps as the dependent variables and the subject’s age, sex, medication dosage, symptoms (PANSS total scores), and global cognitive score (g) as the covariates, with family-wise error correction to compensate for multiple testing. In a supplemental analysis, a MANCOVA was also used to investigate the relationships of the loading coefficients as the dependent variables and each of the cognitive tests as covariates.

## Results

### Subject Demographics

A Student’s two-sample *t*-test showed there were no significant differences between HC and SZ groups with regards to age (*t* = –0.729, *p*-value = 0.467). A Chi-squared test determined that the two groups, HC and SZ, were balanced for gender as well (X-squared = 0.32614, *p*-value = 0.568). The Mood’s median test showed no significant differences between the groups for education level (z = 1.18, *p*-value = 0.24) or for level of occupation (z = 1.26, *p*-value = 0.21) (see Table 1).

### Parallel Independent Component Analysis

**FIGURE 1:**
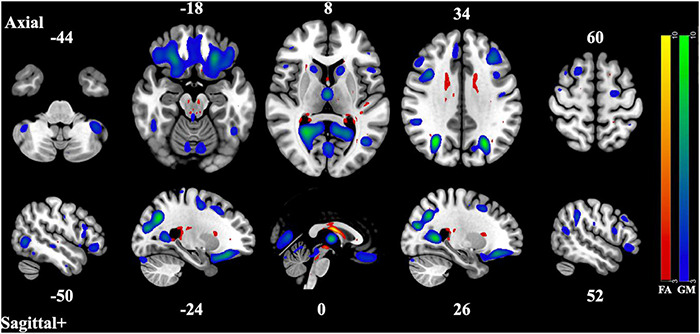
Component Pair 1, Intermodal spatial map highlighting the correlated FA and GM changes that differ significantly (Bonferroni-corrected for multiple comparisons), HC > SZ, z > |3|. pICA correlation between structural networks, *r* = 0.61 (*t* = 9.56, *p* = 2.87 × 10^– 17^). FA cases vs. controls differences, *t* = 3.21, *p* = 0.0016, GM cases vs. controls, *t* = 3.63, *p* = 0.00039. Red-yellow represents the FA group differences, blue-green represents the GM group differences. See Supplementary Figures 1–2 for full axial and sagittal images.

**FIGURE 2:**
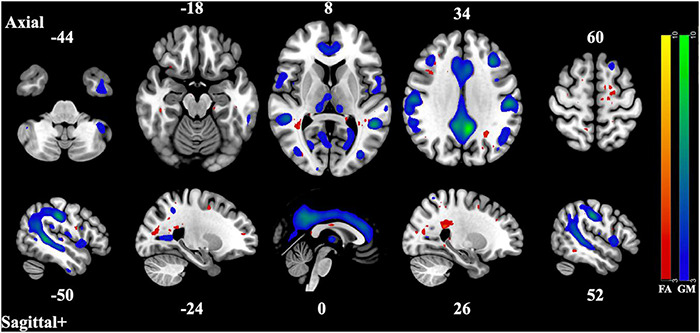
Component Pair 2, Intermodal spatial map highlighting the correlated FA and GM changes that differ significantly (Bonferroni-corrected for multiple comparisons), HC > SZ, z > |3|. pICA correlation between structural networks, *r* = 0.59 (*t* = 9.05, *p* = 6.18 × 10^– 16^). FA cases vs. controls differences, *t* = 2.99, *p* = 0.0032, GM cases vs. controls, *t* = 2.67, *p* = 0.0082. Red-yellow represents the FA group differences, blue-green represents the GM group differences. See Supplementary Figures 3, 4 for full axial and sagittal images.

**FIGURE 3:**
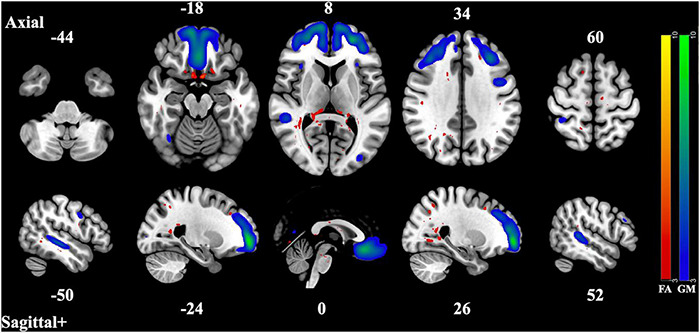
Component Pair 3, Intermodal spatial map highlighting the correlated FA and GM changes that differ significantly (Bonferroni-corrected for multiple comparisons), HC > SZ, z > |3|. pICA correlation between structural networks, *r* = 0.46 (*t* = 6.45, *p* = 1.36 × 10^– 9^). FA cases vs. controls differences, *t* = 3.5, *p* = 0.00053, GM cases vs. controls, *t* = 2.92, *p* = 0.0041. Red-yellow represents the FA group differences, blue-green represents the GM group differences. See Supplementary Figures 5–6 for full axial and sagittal images.

The 12 component model found 6 significantly correlated pairs of FA and GM changes. Of those 6, *t*-tests of the subjects’ loading coefficients determined that 3 pairs had significant group differences (cases vs. controls, HC > SZ, Bonferroni-corrected *p*-value < 0.002) in the correlated patterns of FA/GM changes. Figures 1–3 show the spatial maps of the significantly correlated FA/GM brain regions with significant group differences. Tables 2A–C are an abridged list of the brain regions of GM and WM found in the significantly correlated component pairs. See Supplementary Tables 1–3 for the comprehensive list.

**TABLE 2:** Abridged List of Brain Regions—**(A)** Component Pair 1, **(B)** Component Pair 2, **(C)** Component Pair 3.

GM brain region	|Z|	MNI (x, y, z)	FA brain region	|Z|	MNI (x, y, z)
**(A)**					
Lateral occipital cortex, superior division	11.2	(30, –64, 34)	Thalamus, left	9.6	(–13, –12, 19)
Precunous cortex	9.2	(24, –54, 6)	Caudate, left	8.7	(–15, –15, 21)
Lingual gyrus	8.3	(24, –50, 4)	Thalamus, right	8.3	(16, –21, 19)
Lateral occipital cortex, inferior division	8	(–44, –66, 2)	Planum temporal	6	(–36, –34, 12)
Middle frontal gyrus	7.7	(38, 12, 30)	Retrolenticular part of internal capsule	5.7	(31, –37, 15)
Frontal orbital cortex	7.5	(–24, 30, –18)	Body of corpus callosum	5.7	(5, –11, 28)
Angular gyrus	6.7	(44, –56, 16)	Posterior thalamic radiation, left	5.4	(–31, –39, 11)
Thalamus, right	6.6	(4, –12, 6)	Posterior thalamic radiation, right	5.2	(33, –39, 11)
Thalamus, left	6.4	(–14, 18, 0)	Helschl’s gyrus (H1 and H2)	5.1	(–44, –23, 9)
**(B)**					
Precunous cortex	9.6	(–6, –52, 34)	Middle frontal gyrus	6.3	(36, 17, 31)
Cingulate gyrus, posterior division	9.1	(–8, –48, 34)	Precunous cortex	5.9	(–11, –62, 29)
Angular gyrus	8.8	(–42, –58, 22)	Tapetum, right	5.8	(27, –43, 21)
Postcentral gyrus	7.8	(–52, –24, 36)	Precentral gyrus	5.7	(–15, –16, 62)
Middle frontal gyrus	7.5	(–36, 26, 36)	Supramarginal gyrus, posterior division	5.7	(37, –47, 10)
Superior parietal lobule	6.5	(–30, –50, 44)	Superior frontal gyrus	5.6	(–17, –3, 60)
Middle temporal gyrus	6.3	(50, –36, 4)	Lateral occipital cortex, inferior division	5.3	(33, –80, 9)
Supramarginal gyrus, posterior division	6.2	(50, –40, 8)	Tapetum, left	5.3	(46, –21, 35)
Cingulate gyrus, anterior division	6.2	(–4, 26, 26)	Cingulum (hippocampus), left	5.2	(–15, –80, 30)
**(C)**					
Frontal pole	9.5	(–26, 56, –2)	Subcallosal cortex	8.8	(–5, 9, –19)
Paracingulate gyrus	7.7	(–2, 32, –14)	Thalamus, right	8.1	(15, –33, 12)
Frontal medial cortex	7.4	(2, 34, –14)	Thalamus, left	7.9	(–8, –28, 16)
Middle frontal gyrus	6.5	(42, 22, 24)	Precentral gyrus	7.3	(–40, –1, 41)
Subcallosal cortex	6.1	(–2, 24, –14)	Middle frontal gyrus	6.7	(34, 26, 30)
Middle temporal gyrus, posterior division	5.7	(–52, –26, –6)	Lateral occipital cortex, superior division	6.7	(37, –67, 30)
Middle temporal gyrus, temporoccipital	5.4	(50, –36, 4)	Cingulum (hippocampus) left	6.5	(–16, –39, –5)
Lateral occipital cortex, inferior division	5.1	(–32, –86, 4)	Cingulum (hippocampus) right	5.9	(17, –39, –4)
Angular gyrus	4.3	(–40, –60, 26)	Lingual gyrus	5.7	(29, –56, 2)

*For each component pair, the top brain regions in the spatial map thresholded above |Z| > 3.0 were identified by their Montreal Neurological Institute (MNI) coordinates. See Supplementary Figures 1–6 for the comprehensive list of regions for each component pair.*

### Cognitive Performance

The PCA of the cognitive battery resulted in a primary component that explained 39.9% of the variance. This component was used as a global cognitive measure (g). A Welch’s two sample *t*-test of g showed HC performed significantly better on the cognitive tests than SZ (*t* = 9.987, *p* < 2.2 × 10^–16^).

### Statistical Analysis

**FIGURE 4:**
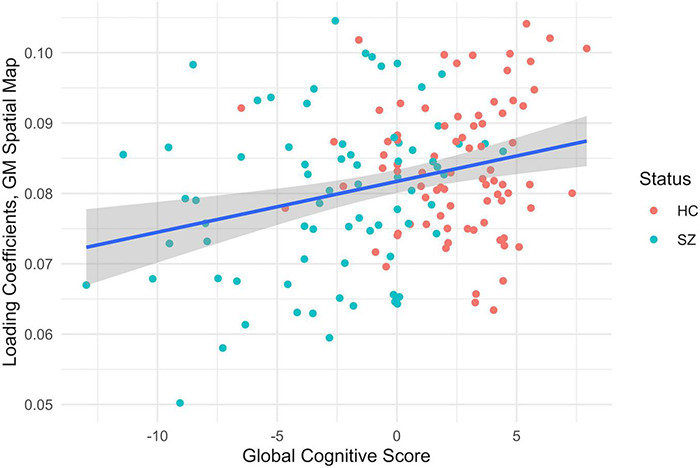
MANCOVA results between the subjects’ gray matter loading coefficients from the second component pair (y-axis) and the global cognitive score (x-axis), *F* = 12.93, *p* < 0.001, corrected for multiple comparisons, healthy controls (HC) in red and patients with schizophrenia in teal (SZ). The gray shading indicates a confidence interval of 0.95.

**FIGURE 5:**
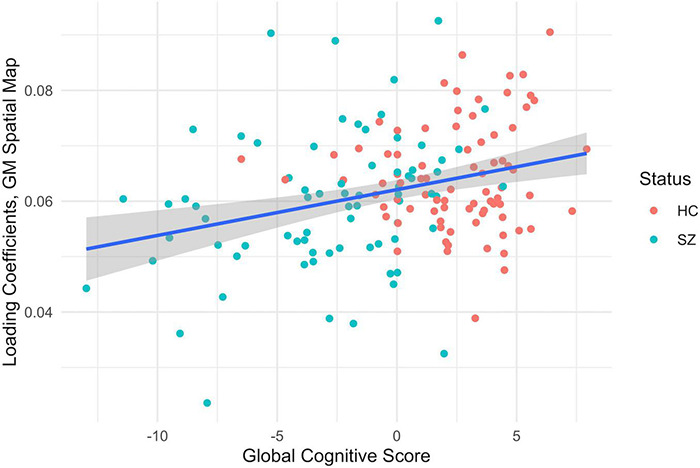
MANCOVA results between the subjects gray matter loading coefficients from the third component pair (y-axis) and the global cognitive score (x-axis), *F* = 9.70, *p* < 0.002, corrected for multiple comparisons, healthy controls (HC) in red and patients with schizophrenia in teal (SZ). The gray shading indicates a confidence interval of 0.95.

The MANCOVA showed the expected significant relationships between the correlated FA/GM brain regions and the subject’s age and sex. No significant relationships were found for medication dosage or symptoms, but there was a weak inverse correlation between the Total Negative Score and the global cognitive scores in patients (*r* = –0.35, *p* = 0.01, Bonferroni-corrected for multiple comparisons). Two of the correlated spatial maps showed significant relationships between the GM differences and the global cognitive score (g), shown in Figures 4, 5. See Supplementary Tables 4–6 for a list the significant relationships found for each of the correlated FA/GM spatial maps. Significant relationships were also found between these same two GM spatial maps and individual cognitive tests, but they did not survive Bonferroni error correction. These were the WTAR Standard Score, the WASI Vocab T-Score, the WASI Verbal T-Score, the WASI Block Design T-Score, the NAB Mazes T-Score, and the Matrics Domain Reasoning and Problem Solving T-Score. See Supplementary Tables 7, 8 for full results.

## Discussion

This multimodal analysis highlighted three unique structural networks in which patients with schizophrenia had significant differences in their patterns of gray and white matter covariance. Gray matter and white matter are often treated as though they are separate and distinct structures, but they are in fact parts of the same neurons. Studying GM and FA together offers the opportunity to see what patterns of covarying GM and FA differ between HC and SZ and how these relationships present themselves behaviorally across the two different groups. While not networks in the neuroanatomical sense, patterns of structural change/difference are often referred to as structural networks. The high incidence of co-location of gray and white matter differences in the results of this study lend support to their behaving in a networked manner, as does the similarity between these structural networks and the functional network differences found in unrelated and independent cohorts used in fMRI and rs-fMRI studies of cognition. The brain regions of correlated covariance identified in each of these component pairs had less gray matter volume and reduced white matter integrity than healthy controls. These networks pull from broad regions of the brain, reflecting the ways in schizophrenia globally impacts the brain.

The first component pair found widespread reduction of gray matter volume in areas such as the lateral occipital cortex, the precuneus cortex, the lingual cortex, the thalamus, the frontal pole, the frontal orbital cortex, the angular gyrus, the supra marginal gyrus, the caudate, as well as areas of the middle frontal gyrus and the frontal orbital cortex. The areas of reduced white matter integrity that were correlated to those gray matter areas were found in the body of the corpus callosum, the thalamus, the caudate, the retrolenticular part of the internal capsule, the posterior thalamic radiations, the superior fronts-occipital fasciculi, as well as the external capsule and subcallosal cortex. The overarching pattern of these regions seems to implicate the front-to-back-to-front connectivity of the brain, approximating control networks seen in functional and resting-state MRI studies (f- and rsfMRI) (Fair et al., 2007; Gratton et al., 2018). These control networks include the fronto-parietal, the cingular-opercular, and the dorsal attentional networks and are responsible for regulating other brain systems such as cognitive control and cognitive flexibility (Cole et al., 2014). Disturbances within this overarching control network would likely cause downstream dysregulation across neural systems (Cole et al., 2014). This particular structural network was the most strongly correlated of the three component pairs, suggesting that the most marked difference between the covarying gray and white matter networks of the two groups lies within this control network.

The second and third component pair showed reduced gray matter volume and less white matter integrity in areas associated with the three subnetworks of the default mode network (DMN) (Andrews-Hanna et al., 2010). The pattern of gray matter volume differences seen in the second component pair resemble those of the midline core subsystem of the DMN. These consist of the precuneus cortex, the posterior cingulate gyrus, the angular gyrus, the postcentral gyrus, the middle frontal gyrus, and the superior and inferior lateral occipital cortex. The correlated covarying white matter areas with reduced integrity were also in tracts and regions that connect these subnetworks, such as the corpus callosum, the middle frontal gyrus, the precuneus cortex, the posterior supramarginal gyrus, and inferior and superior lateral occipital cortex. This midline core subsystem of the DMN is considered a coordinating hub between the other two subsystems (Andrews-Hanna et al., 2010). The third component pair seems to highlight areas of gray matter differences related to the two smaller subsystems of the DMN, the medial temporal lobe subsystem and the dorsal medial prefrontal cortex subsystem, by including the frontal pole, the paracingulate gyrus, the frontal medial cortex, the posterior and temporoccipital middle temporal gyrus, and the inferior lateral occipital cortex. The correlated regions of reduced white matter structural integrity were seen in structures related to the coordination and communication between these subnetworks such as the thalamus, the middle frontal gyrus, the superior lateral occipital cortex, the lingual gyrus, the splenium of the corpus callosum, the posterior cingulate, the temporoccipital middle temporal gyrus as well as the inferior lateral occipital cortex. These two subsystems have been identified as being involved in memory and metacognition, as well as the self-reflective processes typically associated with the DMN (Andrews-Hanna et al., 2010).

In both of these component pairs, the gray matter component loading coefficients were also related to differences in cognitive performance, where loading coefficients of the healthy controls were associated with higher global cognitive scores than patients with schizophrenia. These subnetworks of the DMN have been linked to cognition in several recent studies. In a 2018 fMRI study, researchers established significantly increased activation of subnetworks within the DMN during cognitive task switching and concluded that these networks, normally associated with contextual representation, were recruited when a shift in cognitive focus was required (Smith et al., 2018). Another study in 2020 found that multiple DMN subnetworks were involved in a variety of cognitive tasks (Gordon et al., 2020). A 2021 review of the functional role of the DMN found that it was involved in forms of complex cognition that involve abstract thought and memory (Smallwood et al., 2021). Structural networks map well to functional ones and vice versa (Meier et al., 2016), so deficits in the gray and white matter structural network could be responsible for dysfunction in the functional network, impacting cognitive performance in patients.

Using pICA to look at both of white and gray matter structures simultaneously revealed unique networks of differences in the structural networks in patients with schizophrenia that echo networks found in functional studies. Dysregulation of the functional networks is a common finding within schizophrenia research. Task fMRI studies have found aberrant connectivity both within the frontoparietal network as well as between it and the rest of the brain (Tu et al., 2013) and dysconnectivity with regards to attentional tasks and the frontoparietal network (Roiser et al., 2013). A recent task-based fMRI study found that disrupted frontoparietal control networks as well as dysconnectivity within the DMN were related to metacognitive deficits, demonstrating that global dysfunction of these networks interferes with overarching cognitive processes (Jia et al., 2020). Disrupted DMN network activation/deactivation has been long associated with schizophrenia (Hu et al., 2017), and a dynamic connectivity study has shown that patients have reduced connectivity between the DMN subnetworks (Du et al., 2016).

Many of the brain regions of covarying GM and FA found in the three component pairs of this study are also involved in the cortical-subcortical-cerebellar circuit considered critical for appropriate cognitive performance (Andreasen et al., 1996). Dysregulation of the DMN networks as well as the thalamus, cerebellum, and the inferior frontal gyrus (IFG) in the patients was associated with poor cognitive performance (Matsuo et al., 2013). A 2018 study found that reduced gray matter volume in the insula, inferior parietal cortex, middle temporal cortex, and cerebellum in patients were related to poor cognitive performance (Banaj et al., 2018). A study in first episode patients found that reduced prefrontal and temporo-parietal gray matter volume was significantly correlated with poor cognitive performance (Minatogawa-Chang et al., 2009).

In this study, only the gray matter volume differences were found to be related to poor cognitive performance, differentiating it from the white matter integrity differences. A study done in 2018 investigated gray and white matter volumes independently also found that lower cognitive performance in patients was correlated with brain regions that showed less gray matter volume than healthy controls (Banaj et al., 2018). The affected gray matter regions in that study were similar to the ones found in this study; the inferior parietal cortex, the insula, the middle temporal cortex, and the cerebellum.

The weak inverse correlation between the Total Negative Score and the global cognitive scores in patients is a not uncommon (although inconsistent) finding and has been linked by several studies to the “difficulty in abstract thinking” question of the PANSS negative sub scale. This measure is thought to have some overlap with cognitive domains, but not enough to consider them collinear (Cruz et al., 2013; Bagney et al., 2015).

### Next Steps

To further our understanding of the relationships between the covarying gray and white matter areas identified in the component pairs, a tractography study could shed more light on why these particular patterns are covarying together. A longitudinal study from prodromal through a chronic state could better quantify the causal relationship between the changes within the structural networks and their relationship with the typical decline in cognitive performance.

### Limitations

Since this is a cross-sectional study, there are limitations regarding the interpretations of the relationships between the covarying structural networks and cognitive performance.

The decision to create a summary measure of cognitive performance was done to reduce the number of statistical tests required, minimizing the likelihood of Type I errors. This was done with the understanding that schizophrenia is commonly characterized by a pattern of generalized cognitive impairment.

All patients in this study were taking some form of anti-psychotic medication.

## Conclusion

This multimodal investigation of the correlated patterns of structural network covariance highlighted three unique networks of decreased gray matter volume and reduced white matter integrity in patients with schizophrenia, as well as a relationship between these networks and their diminished cognitive performance consistently across both subject groups. These networks show similarities with frontoparietal control networks and subnetworks of the DMN and could be the structural underpinnings for the well-established disruptions found in schizophrenia. These structural networks are also implicated in the cortical-subcortical-cerebellar circuit, the dysregulation of which is also associated with poor global cognitive performance.

## Data Availability Statement

Publicly available datasets were analyzed in this study. This data can be found here: https://www.mrn.org/common/cobre-phase-3.

## Ethics Statement

The studies involving human participants were reviewed and approved by the Georgia State University. The patients/participants provided their written informed consent to participate in this study.

## Author Contributions

DJ, EZ, VC, and JT contributed to the design and implementation of the research, to the analysis of the results and to the writing of the manuscript. All authors contributed to the article and approved the submitted version.

## Conflict of Interest

The authors declare that the research was conducted in the absence of any commercial or financial relationships that could be construed as a potential conflict of interest.

## Publisher’s Note

All claims expressed in this article are solely those of the authors and do not necessarily represent those of their affiliated organizations, or those of the publisher, the editors and the reviewers. Any product that may be evaluated in this article, or claim that may be made by its manufacturer, is not guaranteed or endorsed by the publisher.
