# Supplementary material for: Cognitive Implications of Correlated Structural Network Changes in Schizophrenia
Source: Front Integr Neurosci. 2022 Jan 20;15:755069. doi: 10.3389/fnint.2021.755069 (PMC8811375; doi:10.3389/fnint.2021.755069)
Supplement: Supplementary file 1 [file Data_Sheet_1.docx]

Supplementary Material

# Supplementary Tables:

**Supplementary Table 1A: Component Pair 1, Grey Matter Volume Differences -** Areas of GM differences identified in the spatial maps of Component Pair 1, (HC>SZ). Significant regions were thresholded above |Z|>3.0 and were identified by their Montreal Neurological Institute (MNI) coordinates.

**1A)**

| **Area** | **Volume (cc)** | **Max Value** | **MNI (x, y, z)** |
| --- | --- | --- | --- |
| **Lateral Occipital Cortex, superior division** | 35 | 11.2 | (30, -64, 34) |
| **Precuneus Cortex** | 13.3 | 9.2 | (24, -54, 6) |
| **Lingual Gyrus** | 6.6 | 8.3 | (24, -50, 4) |
| **Lateral Occipital Cortex, inferior division** | 3.1 | 8 | (-44, -66, 2) |
| **Middle Frontal Gyrus** | 14.9 | 7.7 | (38, 12, 30) |
| **Frontal Orbital Cortex** | 41.1 | 7.5 | (-24, 30, -18) |
| **Angular Gyrus** | 2.6 | 6.7 | (44, -56, 16) |
| **Thalamus, Left** | 2.7 | 6.4 | (-14, 18, 0) |
| **Supramarginal Gyrus, posterior division** | 4.5 | 6.1 | (38, -42, 40) |
| **Caudate, Left** | 2.1 | 5.6 | (-14, 16, -4) |
| **Superior Frontal Gyrus** | 2.8 | 5.2 | (24, 14, 48) |
| **Frontal Pole** | 2.8 | 5 | (40, 38, -16) |
| **Cerebellum, Left Crus II** | 2.6 | 5 | (-40, -48, -44) |
| **Cingulate Gyrus, posterior division** | 2.4 | 4.8 | (-12, -48, 0) |
| **Postcentral Gyrus** | 2.6 | 4.7 | (56, -18, 26) |
| **Caudate, Right** | 2.2 | 4.7 | (14, 18, -2) |
| **Cerebellum, Left VI** | 2.8 | 4.6 | (-8, -76, -20) |
| **Frontal Medial Cortex** | 3.5 | 4.5 | (-2, 44, -22) |
| **Cerebellum, Right Crus I** | 3.3 | 4.5 | (44, -52, -34) |
| **Cerebellum, Left Crus I** | 1.5 | 4.5 | (-44, -54, -36) |
| **Precentral Gyrus** | 5 | 4.4 | (-40, -16, 40) |
| **Inferior Temporal Gyrus, temporooccipital part** | 1.2 | 4.3 | (-46, -54, -18) |
| **Paracingulate Gryrus** | 4.8 | 3.9 | (6, 22, 44) |
| **Cerebellum, Right VI** | 1.5 | 3.9 | (8, -76, -20) |

**Supplementary Table 1B: Component Pair 1, White Matter Structural Differences -** Areas of FA differences identified in the spatial maps of Component Pair 1, (HC>SZ). Significant regions were thresholded above |Z|>3.0 and were identified by their Montreal Neurological Institute (MNI) coordinates.

**1B)**

| **Area** | **volume (cc)** | **Max Value** | **MNI (x, y, z)** |
| --- | --- | --- | --- |
| **Thalamus, Left** | 5.1 | 9.6 | (-13, -12, 19) |
| **Caudate, Left** | 5.4 | 8.7 | (-15, -15, 21) |
| **Thalamus, Right** | 8.4 | 8.3 | (16, -21, 19) |
| **Planum Temporale** | 1 | 6 | (-36, -34, 12) |
| **Retrolenticular part of Internal Capsule Right** | 2 | 5.7 | (31, -37, 15) |
| **Body of Corpus Callosum** | 3.3 | 5.7 | (5, -11, 28) |
| **Posterior Thalamic Radiation (include optic radiation) Left** | 1.9 | 5.4 | (-31, -39, 11) |
| **Posterior Thalamic Radiation (include optic radiation) Right** | 1.7 | 5.2 | (33, -39, 11) |
| **Superior Fronto-occipital Fasciculus Right** | 3.3 | 5 | (21, 10, 24) |
| **External Capsule Left** | 3 | 4.7 | (-25, 11, 13) |
| **Superior Fronto-occipital Fasciculus Left** | 4.1 | 4.2 | (-20, 9, 24) |
| **Subcallosal Cortex** | 1.1 | 3.7 | (5, 8, -19) |
| **Precentral Gyrus** | 1.2 | 3.6 | (-16, -15, 64) |

**Supplementary Table 2A: Component Pair 2, Grey Matter Volume Differences -** Areas of GM differences identified in the spatial maps of Component Pair 2, (HC>SZ). Significant regions were thresholded above |Z|>3.0 and were identified by their Montreal Neurological Institute (MNI) coordinates.

**2A)**

| **Area** | **volume (cc)** | **Max Value** | **MNI (x, y, z)** |
| --- | --- | --- | --- |
| **Precuneus Cortex** | 24.2 | 9.6 | (-6, -52, 34) |
| **Cingulate Gyrus, posterior division** | 26.2 | 9.1 | (-8, -48, 34) |
| **Angular Gyrus** | 19.3 | 8.8 | (-42, -58, 22) |
| **Postcentral Gyrus** | 20 | 7.8 | (-52, -24, 36) |
| **Middle Frontal Gyrus** | 10.3 | 7.5 | (-36, 26, 36) |
| **Superior Parietal Lobule** | 14.6 | 6.5 | (-30, -50, 44) |
| **Middle Temporal Gyrus** | 3.7 | 6.3 | (50, -36, 4) |
| **Supramarginal Gyrus, posterior division** | 11.2 | 6.2 | (50, -40, 8) |
| **Cingulate Gyrus, anterior division** | 10.7 | 6.2 | (-4, 26, 26) |
| **Lateral Occipital Cortex, superior division** | 2 | 5.9 | (-44, -62, 30) |
| **Supramarginal Gyrus, anterior division** | 5.6 | 5.9 | (38, -34, 44) |
| **Lateral Occipital Cortex, inferior division** | 3.3 | 5.5 | (-36, -80, 10) |
| **Thalamus, Right** | 2.4 | 5.5 | (12, -28, 4) |
| **Thalamus, Left** | 1.2 | 4.9 | (-12, -30, 4) |
| **Paracingulate Gyrus** | 2.3 | 4.9 | (-6, 42, 20) |
| **Inferior Frontal Gyrus, pars opercularis** | 1 | 4.6 | (52, 10, 2) |
| **Intracalcarine Cortex** | 2.6 | 4.2 | (-20, -70, 6) |
| **Precentral Gyrus** | 1.1 | 4.1 | (-56, 6, 6) |
| **Frontal Operculum Cortex** | 4.6 | 4.1 | (50, 14, 0) |
| **Cerebellum, Left Crus II** | 1.7 | 4.1 | (-42, -50, -44) |
| **Frontal Orbital Cortex** | 1.6 | 4 | (-42, 26, -12) |
| **Central Opercular Cortex** | 2.2 | 4 | (60, -12, 14) |
| **Superior Frontal Gyrus** | 2.7 | 3.7 | (-20, 22, 58) |

**Supplementary Table 2B: Component Pair 2, White Matter Structural Differences -** Areas of FA differences identified in the spatial maps of Component Pair 2, (HC>SZ). Significant regions were thresholded above |Z|>3.0 and were identified by their Montreal Neurological Institute (MNI) coordinates.

**2B)**

| **Area** | **volume (cc)** | **Max Value** | **MNI (x, y, z)** |
| --- | --- | --- | --- |
| **Middle Frontal Gyrus** | 7.6 | 6.3 | (36, 17, 31) |
| **Precuneus Cortex** | 5.1 | 5.9 | (-11, -62, 29) |
| **Tapetum, Right** | 2.6 | 5.8 | (27, -43, 21) |
| **Precentral Gyrus** | 6.9 | 5.7 | (-15, -16, 62) |
| **Supramarginal Gyrus, posterior division** | 8.7 | 5.7 | (37, -47, 10) |
| **Superior Frontal Gyrus** | 14.2 | 5.6 | (-17, -3, 60) |
| **Lateral Occipital Cortex, inferior division** | 2.1 | 5.5 | (33, -80, 9) |
| **Postcentral Gyrus** | 3.3 | 5.4 | (46, -21, 35) |
| **Cuneal Cortex** | 1.6 | 5.3 | (-15, -80, 30) |
| **Lateral Occipital Cortex, superior division** | 7.4 | 5.3 | (42, -60, 23) |
| **Tapetum, Left** | 1.9 | 5.3 | (-25, -45, 21) |
| **Cingulum (hippocampus) L** | 1.9 | 5.2 | (-19, -29, -16) |
| **Heschl's Gyrus (includes H1 and H2)** | 1.3 | 5.1 | (-46, -25, 3) |
| **Body of Corpus Callosum** | 1 | 4.7 | (9, -18, 28) |
| **Posterior Thalaminc Radiation (include optic radiation) R** | 1.3 | 4.5 | (36, -47, 14) |
| **Angular Gyrus** | 2 | 4.3 | (43, -45, 18) |
| **Lingual Gyrus** | 1.8 | 4.3 | (-25, -56, 4) |
| **Subcallosal Cortex** | 1.5 | 4 | (-3, 12, -19) |
| **Paracingulate Gyrus** | 1.9 | 4 | (-11, 8, 37) |
| **Superior Longitudinal Fasciculus** | 1 | 3.9 | (-45, -41, 5) |
| **Cingulate Gyrus, anterior division** | 1.4 | 3.6 | (11, 40, 6) |

**Supplementary Table 3A: Component Pair 3, Grey Matter Volume Differences -** Areas of GM differences identified in the spatial maps of Component Pair 3, (HC>SZ). Significant regions were thresholded above |Z|>3.0 and were identified by their Montreal Neurological Institute (MNI) coordinates.

**3A)**

| **Area** | **volume (cc)** | **Max Value** | **MNI (x, y, z)** |
| --- | --- | --- | --- |
| **Frontal Pole** | 82.8 | 9.5 | (-26, 56, -2) |
| **Paracingulate Gyrus** | 21.1 | 7.7 | (-2, 32, -14) |
| **Frontal Medial Cortex** | 13.8 | 7.4 | (2, 34, -14) |
| **Middle Frontal Gyrus** | 6.9 | 6.5 | (42, 22, 24) |
| **Subcallosal Cortex** | 2.7 | 6.1 | (-2, 24, -14) |
| **Middle Temporal Gyrus, posterior division** | 3.5 | 5.7 | (-52, -26, -6) |
| **Middle Temporal Gyrus, temporooccipital part** | 5.9 | 5.4 | (50, -36, 4) |
| **Lateral Occipital Cortex, inferior division** | 2.5 | 5.1 | (-32, -86, 4) |
| **Superior Parietal Lobule** | 1.5 | 3.7 | (36, -40, 58) |
| **Precuneus Cortex** | 1.2 | 3.2 | (6, -64, 16) |

**Supplementary Table 3B: Component Pair 3, White Matter Structural Differences -** Areas of FA differences identified in the spatial maps of Component Pair 3, (HC>SZ). Significant regions were thresholded above |Z|>3.0 and were identified by their Montreal Neurological Institute (MNI) coordinates.

**3B)**

| **Area** | **volume (cc)** | **Max Value** | **MNI (x, y, z)** |
| --- | --- | --- | --- |
| **Subcallosal Cortex** | 4.4 | 8.8 | (-5, 9, -19) |
| **Thalamus, Right** | 4.4 | 8.1 | (15, -33, 12) |
| **Thalamus, Left** | 4.1 | 7.9 | (-8, -28, 16) |
| **Precentral Gyrus** | 9.1 | 7.3 | (-40, -1, 41) |
| **Middle Frontal Gyrus** | 7.9 | 6.7 | (34, 26, 30) |
| **Lateral Occipital Cortex, superior division** | 9.8 | 6.7 | (37, -67, 30) |
| **Cingulum (hippocampus) R** | 2.8 | 5.9 | (17, -39, -4) |
| **Lingual Gyrus** | 10.3 | 5.7 | (29, -56, 2) |
| **Splenium of Corpus Callosum** | 1.2 | 5.1 | (24, -51, 11) |
| **Supramarginal Gyrus, posterior division** | 1.5 | 5 | (-42, -43, 36) |
| **Cingulate Gyrus, posterior division** | 1.4 | 4.9 | (11, -40, 39) |
| **Frontal Orbital Cortex** | 2.9 | 4.9 | (-17, 15, -18) |
| **Tapetum L** | 1.7 | 4.9 | (-25, -45, 21) |
| **Middle Temporal Gyrus, temporooccipital part** | 1 | 4.9 | (-50, -53, -3) |
| **Lateral Occipital Cortex, inferior division** | 1.4 | 4.8 | (35, -81, 3) |
| **Frontal Pole** | 1.3 | 4.8 | (26, 37, 31) |
| **Superior Frontal Gyrus** | 1.7 | 4.7 | (13, 15, 59) |
| **Angular Gyrus** | 1 | 4.4 | (-41, -55, 35) |
| **Postcentral Gyrus** | 2.7 | 4.4 | (-26, -37, 55) |
| **Precuneus Cortex** | 1.3 | 4.4 | (-11, -69, 47) |

**Supplementary Table 4: Component Pair 1, Significant Relationships -** All p-values Bonferroni corrected for multiple comparisons.

| **Component Pair 1** | ***p*** | **t** | **F** | **r** | **df** |
| --- | --- | --- | --- | --- | --- |
| **pICA Correlation** | 2.87x10^-17 | 9.56 | — | 0.61 | 10 |
| **Cases v Controls (FA)** | 0.0016 | 3.21 | — | **—** | 154 |
| **Cases v Controls (GM)** | 0.00039 | 3.63 | — | — | 154 |
| **Sex (FA)** | 0.00009 | — | 12.58 | — | 144 |
| **Age (FA)** | < 0.001 | — | 132.54 | — | 144 |
| **Age (GM)** | < 0.001 | — | 86.84 | — | 144 |

**Supplementary Table 5: Component Pair 2, Significant Relationships -** All p-values Bonferroni corrected for multiple comparisons.

| **Component Pair 2** | ***p*** | **t** | **F** | **r** | **df** |
| --- | --- | --- | --- | --- | --- |
| **pICA Correlation** | 6.18x10^-16 | 9.05 | — | 0.59 | 10 |
| **Cases v Controls (FA)** | 0.0032 | 2.99 | — | **—** | 154 |
| **Cases v Controls (GM)** | 0.0082 | 2.67 | — | — | 154 |
| **Sex (FA)** | Interaction | — | — | — | 144 |
| **Sex (GM)** | 0.004 | — | 8.49 | — | 144 |
| **Age (FA)** | < 0.001 | — | 11.18 | — | 144 |
| **Age (GM)** | < 0.001 | — | 103.19 | — | 144 |
| **g (GM)** | 0.002 | — | 9.70 | — | 144 |

**Supplementary Table 6: Component Pair 3, Significant Relationships -** All p-values Bonferroni corrected for multiple comparisons.

| **Component Pair 3** | ***p*** | **t** | **F** | **r** | **df** |
| --- | --- | --- | --- | --- | --- |
| **pICA Correlation** | 1.36x10^-9 | 6.45 | — | 0.46 | 10 |
| **Cases v Controls (FA)** | 0.00053 | 3.5 | — | **—** | 154 |
| **Cases v Controls (GM)** | 0.0041 | 2.92 | — | — | 154 |
| **Sex (GM)** | < 0.001 | — | 11.63 | — | 144 |
| **Age (GM)** | < 0.001 | — | 40.39 | — | 144 |
| **g (GM)** | < 0.001 | — | 12.93 | — | 144 |

**Supplementary Table 7: Component Pair 2-** MANCOVA results for individual cognitive tests, uncorrected relationships with grey matter volume differences.

| **Cognitive Tests** | **F** | ***p*** | **df** |
| --- | --- | --- | --- |
| **Wechsler Test of Adult Reading** | 14.215 | 0.0004 | 144 |
| **Wechsler Abbreviated Scale of Intelligence - Vocabulary** | 8.008 | 0.005 | 144 |
| **Wechsler Abbreviated Scale of Intelligence - Verbal Score** | 8.371 | 0.004 | 144 |
| **Wechsler Abbreviated Scale of Intelligence - Block Design** | 14.290 | 0.0002 | 144 |
| **Neuropsychological Assessment Battery Mazes Score** | 7.873 | 0.006 | 144 |
| **Matrics Domain Reason and Problem Solving** | 7.376 | 0.007 | 144 |

**Supplementary Table 8: Component Pair 3-** MANCOVA results for individual cognitive tests, uncorrected relationships with grey matter volume differences.

| **Cognitive Tests** | **F** | ***p*** | **df** |
| --- | --- | --- | --- |
| **Wechsler Test of Adult Reading** | 12.940 | 0.0002 | 144 |
| **Wechsler Abbreviated Scale of Intelligence - Vocabulary** | 6.979 | 0.009 | 144 |
| **Wechsler Abbreviated Scale of Intelligence - Verbal Score** | 8.509 | 0.004 | 144 |
| **Wechsler Abbreviated Scale of Intelligence - Block Design** | 13.549 | 0.0003 | 144 |

## Supplementary Figures

## Supplementary Figure 1): Component Pair 1 full axial, Intermodal spatial map highlighting the correlated FA and GM changes that differ significantly (Bonferroni-corrected for multiple comparisons), HC>SZ, z > |3|. pICA correlation between structural networks, r = 0.61 (t = 9.56, p = 2.87x10^-17).
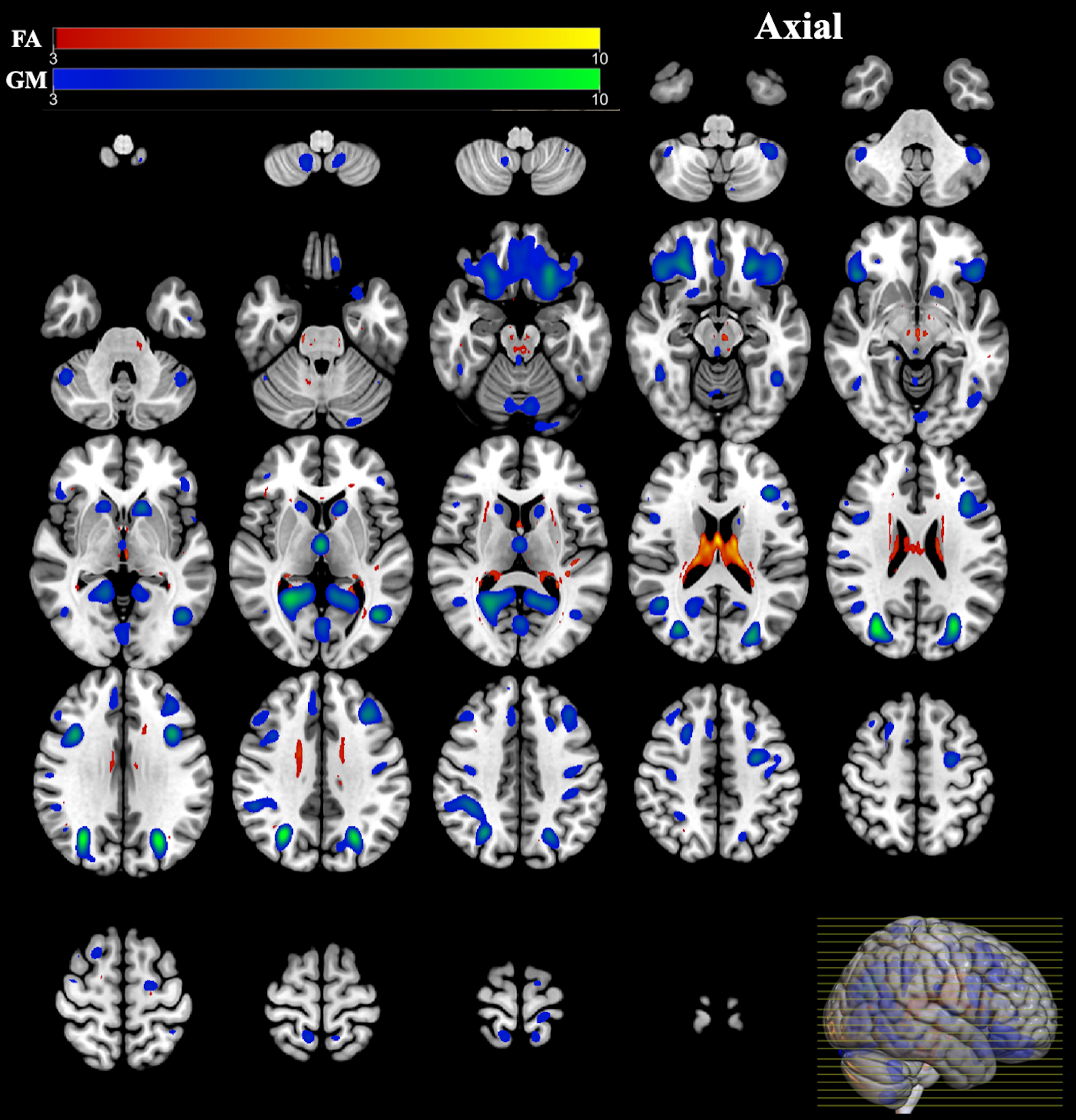
 FA cases vs controls differences, t = 3.21, p = 0.0016, GM cases vs controls, t = 3.63, p = 0.00039. Red-yellow represents the FA group differences, blue-green represents the GM group differences. Brain slice labels: -64, -58, -52, -46, -40; -34, -28, -20, -14, -8; -2, 4, 10, 18, 24; 30, 36, 42, 48, 54; 62, 68, 74, 80.

## Supplementary Figure 2): Component Pair 1 full sagittal+, Intermodal spatial map highlighting the correlated FA and GM changes that differ significantly (Bonferroni-corrected for multiple comparisons), HC>SZ, z > |3|. pICA correlation between structural networks, r = 0.61 (t = 9.56, p = 2.87x10^-17). FA cases
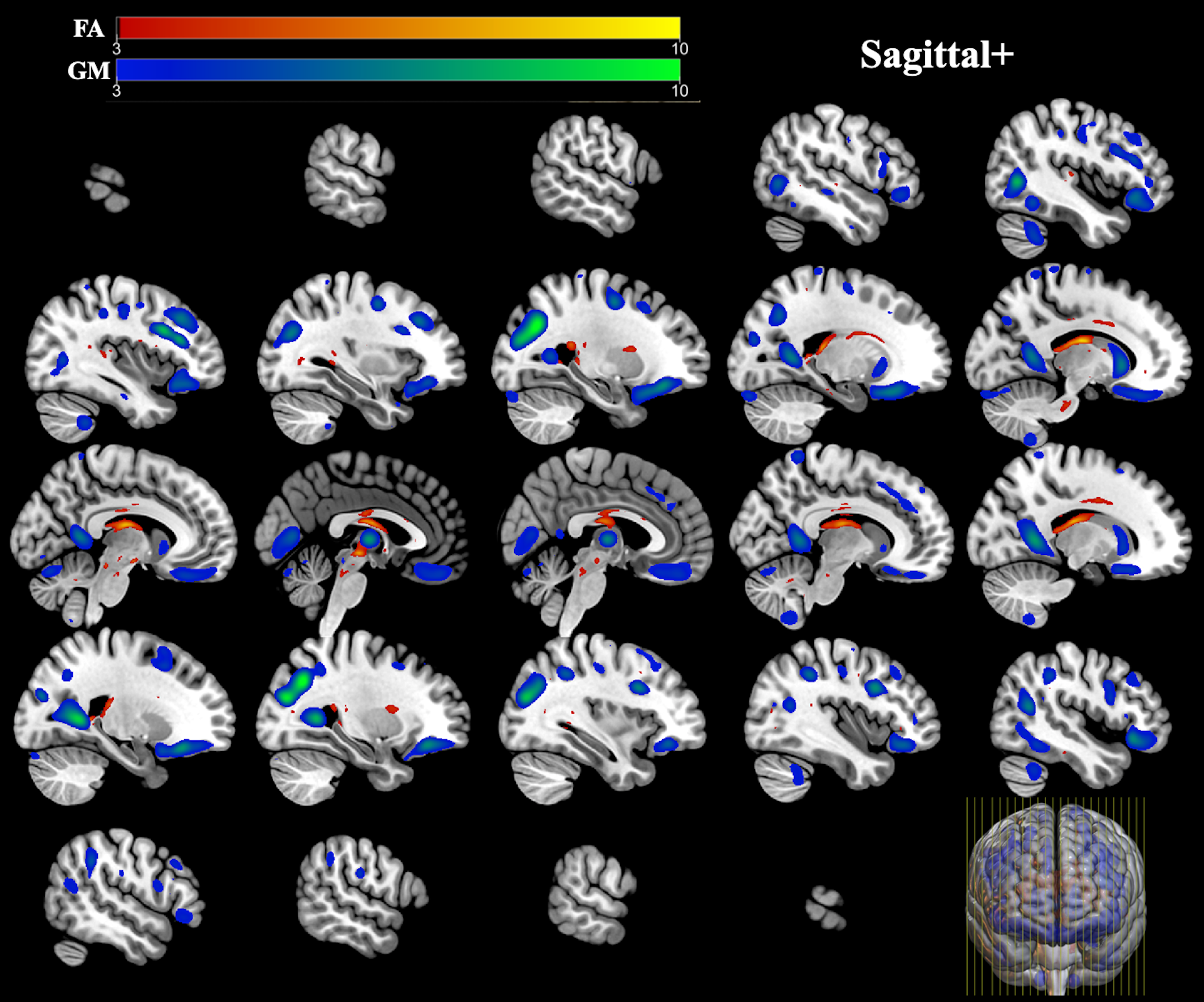
 vs controls differences, t = 3.21, p = 0.0016, GM cases vs controls, t = 3.63, p = 0.00039. Red-yellow represents the FA group differences, blue-green represents the GM group differences. Brain slice labels: -70, -64, -58, -50, -44; -38, -32, -26, -20, -14; -8, -2, 4, 10, 16; 22, 28, 34, 40, 46; 52, 58, 64, 70.

## Supplementary Figure 3): Component Pair 2 full axial, Intermodal spatial map highlighting the correlated FA and GM changes that differ significantly (Bonferroni-corrected for multiple comparisons), HC>SZ, z > |3|. pICA correlation between structural networks, r = 0.59 (t = 9.05, p = 6.18x10^-16). FA cases
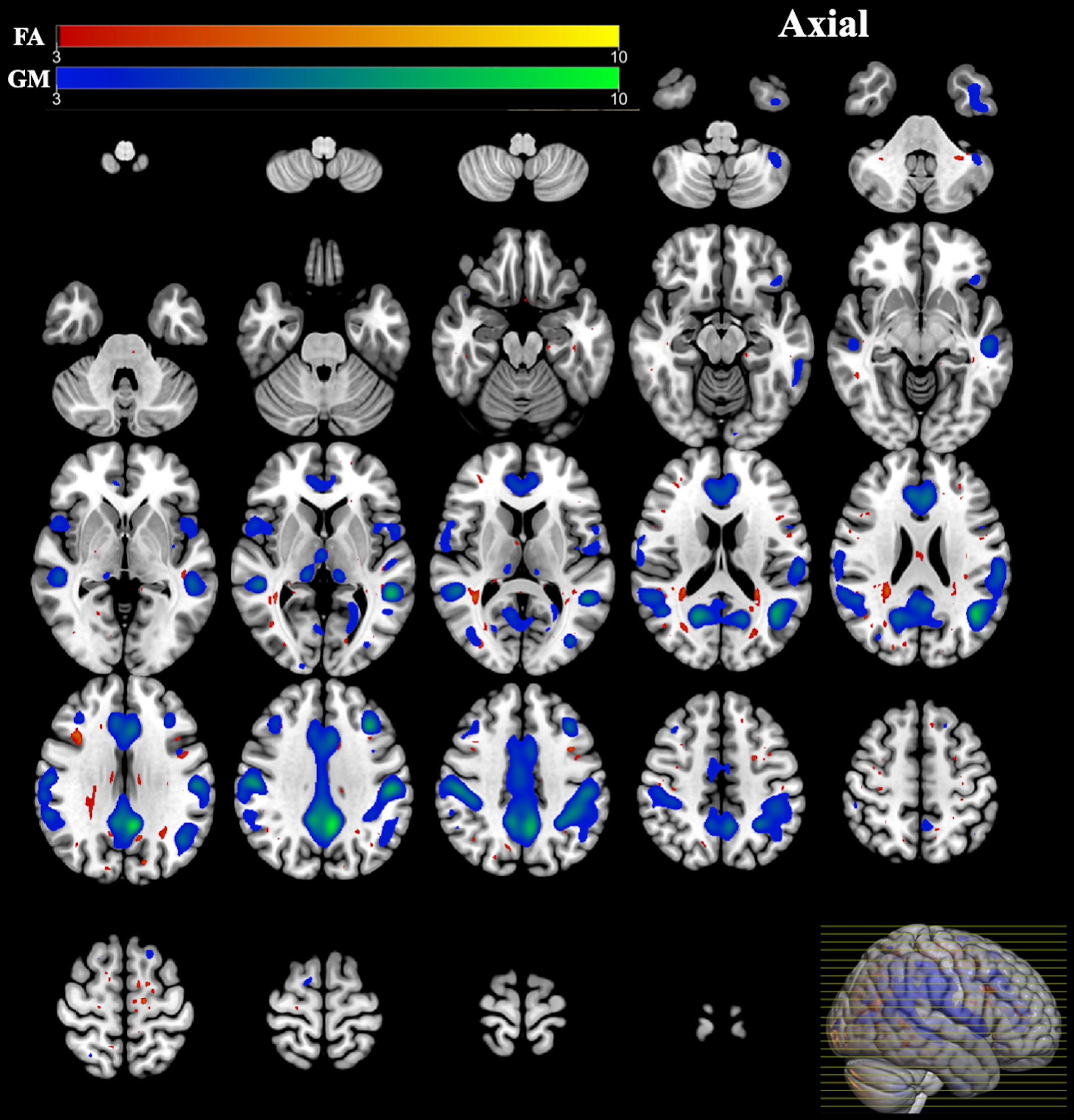
vs controls differences, t = 2.99, p = 0.0032, GM cases vs controls, t = 2.67, p = 0.0082. Red-yellow represents the FA group differences, blue-green represents the GM group differences. Brain slice labels: -64, -58, -52, -46, -40; -34, -28, -20, -14, -8; -2, 4, 10, 18, 24; 30, 36, 42, 48, 54; 62, 68, 74, 80.

## Supplementary Figure 4): Component Pair 2 full sagittal+, Intermodal spatial map highlighting the correlated FA and GM changes that differ significantly (Bonferroni-corrected for multiple comparisons), HC>SZ, z > |3|. pICA correlation between structural networks, r = 0.59 (t = 9.05, p = 6.18x10^-16). FA cases
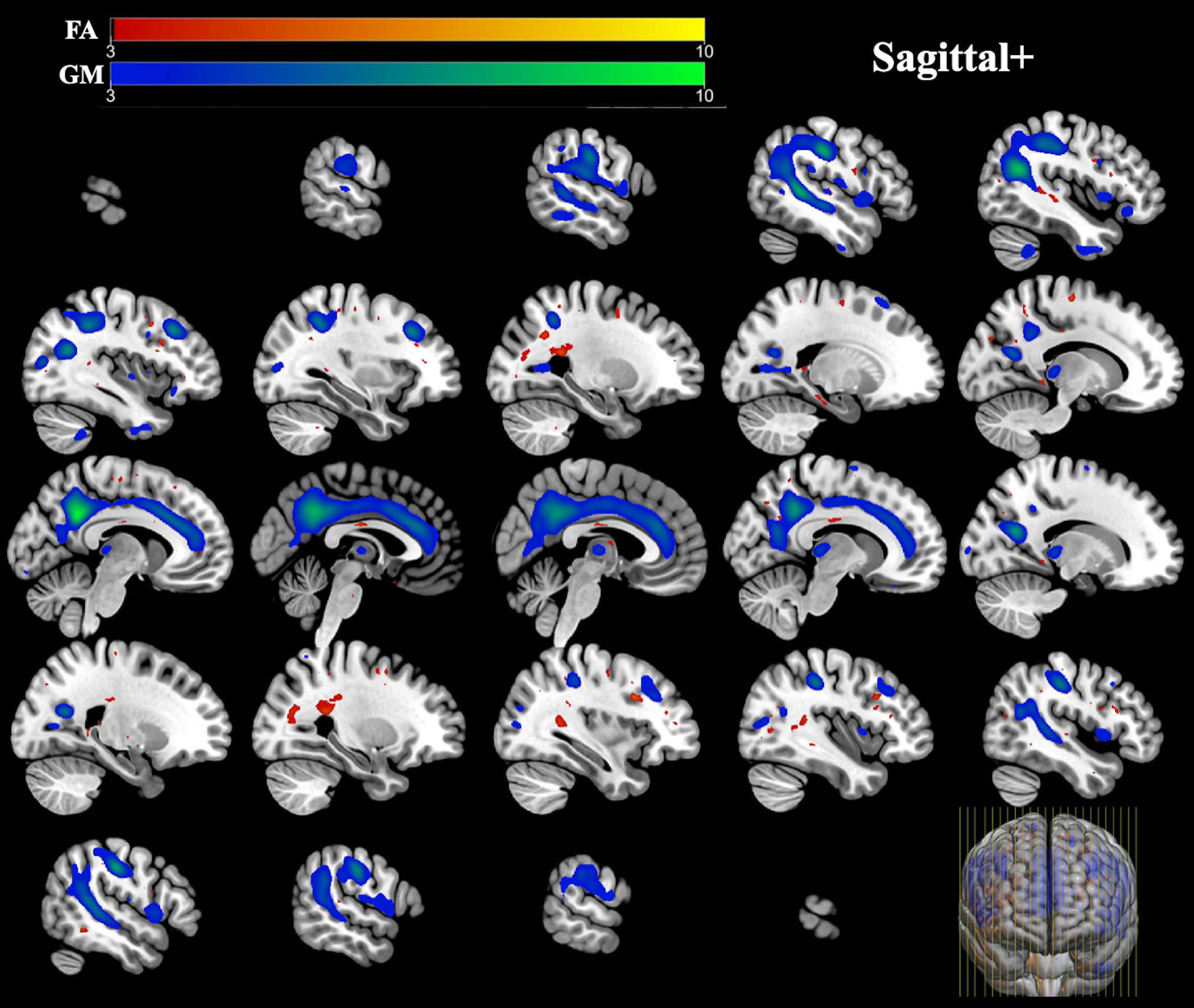
 vs controls differences, t = 2.99, p = 0.0032, GM cases vs controls, t = 2.67, p = 0.0082. Red-yellow represents the FA group differences, blue-green represents the GM group differences. Brain slice labels: -70, -64, -58, -50, -44; -38, -32, -26, -20, -14; -8, -2, 4, 10, 16; 22, 28, 34, 40, 46; 52, 58, 64, 70.

## Supplementary Figure 5): Component Pair 3 full axial, Intermodal spatial map highlighting the correlated FA and GM changes that differ significantly (Bonferroni-corrected for multiple comparisons), HC>SZ, z > |3|. pICA correlation between structural networks, r = 0.46 (t = 6.45, p = 1.36x10^-9). FA cases v
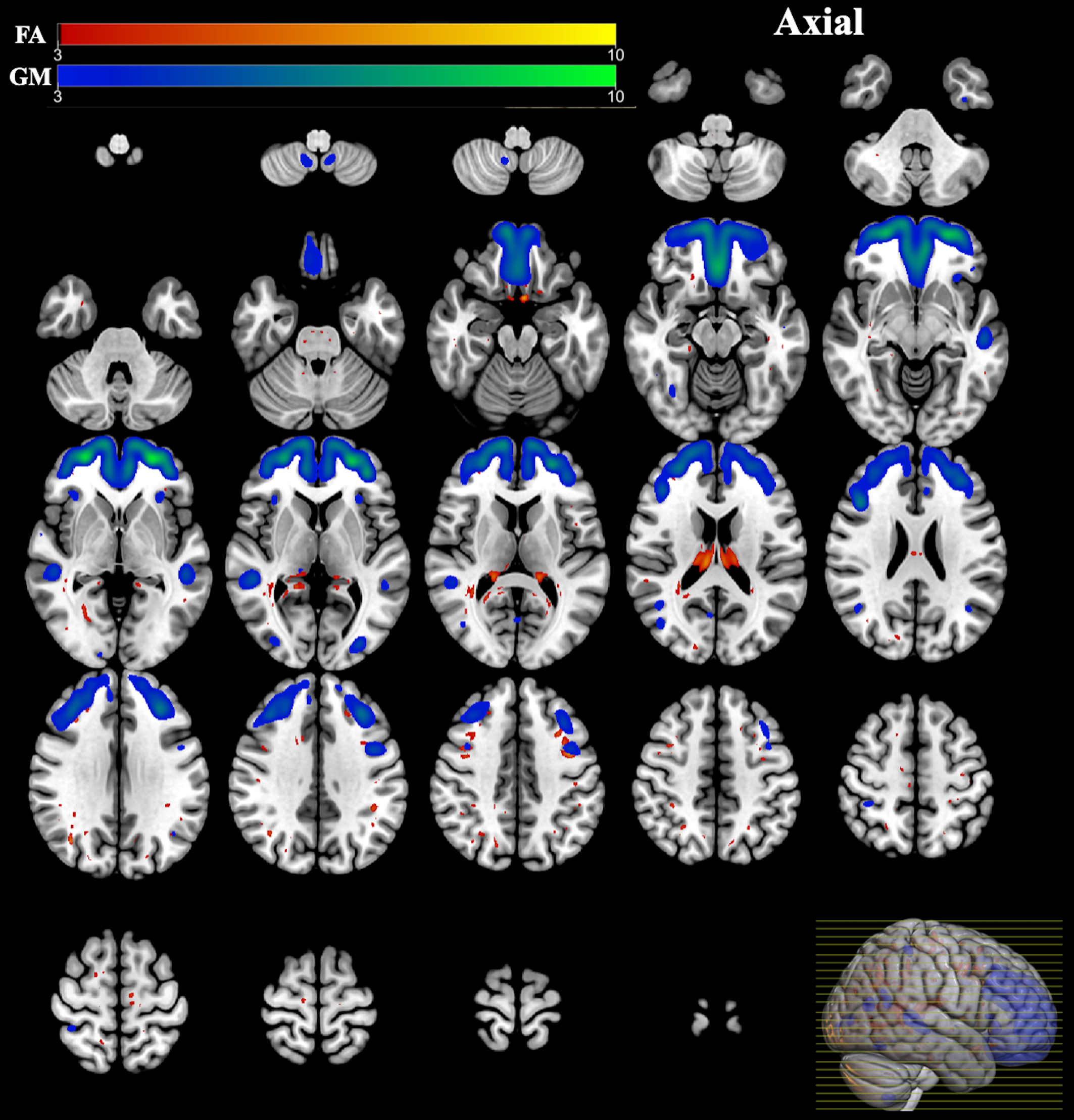
s controls differences, t = 3.5, p = 0.00053, GM cases vs controls, t = 2.92, p = 0.0041. Red-yellow represents the FA group differences, blue-green represents the GM group differences. Brain slice labels: -64, -58, -52, -46, -40; -34, -28, -20, -14, -8; -2, 4, 10, 18, 24; 30, 36, 42, 48, 54; 62, 68, 74, 80.

## Supplementary Figure 6): Component Pair 3 full sagittal+, Intermodal spatial map highlighting the correlated FA and GM changes that differ significantly (Bonferroni-corrected for multiple comparisons), HC>SZ, z > |3|. pICA correlation between structural networks, r = 0.46 (t = 6.45, p = 1.36x10^-9). FA cases
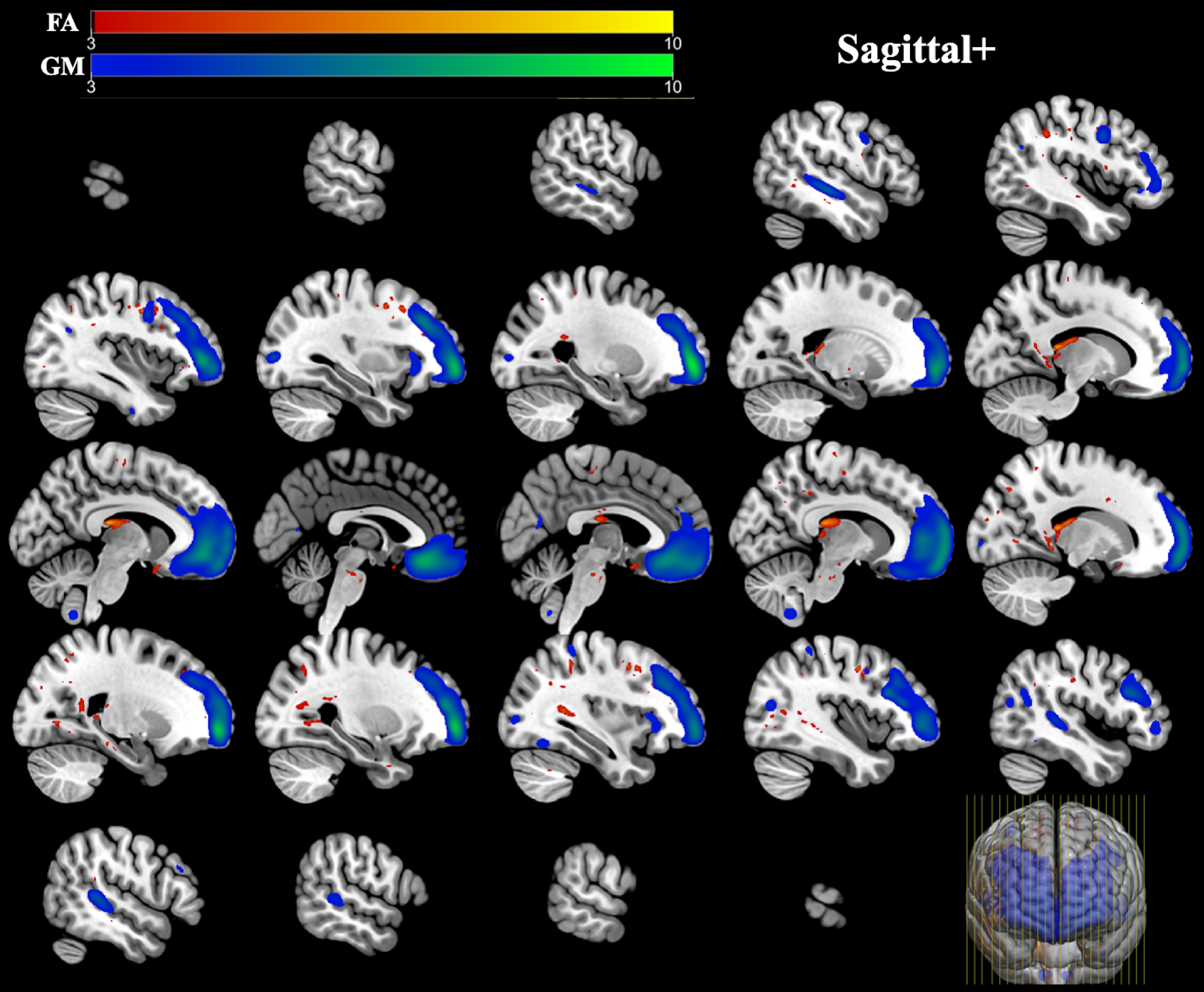
 vs controls differences, t = 3.5, p = 0.00053, GM cases vs controls, t = 2.92, p = 0.0041. Red-yellow represents the FA group differences, blue-green represents the GM group differences. Brain slice labels: -70, -64, -58, -50, -44; -38, -32, -26, -20, -14; -8, -2, 4, 10, 16; 22, 28, 34, 40, 46; 52, 58, 64, 70.
